# Reply to: “Research on agroforestry systems and biodiversity conservation: what can we conclude so far and what should we improve?” by Boinot et al. 2022

**DOI:** 10.1186/s12862-022-02016-7

**Published:** 2022-05-18

**Authors:** Anne-Christine Mupepele, Carsten F. Dormann

**Affiliations:** 1 Nature Conservation and Landscape Ecology, University of Freiburg, Tennenbacherstr. 4, 79106 Freiburg, Germany; 2 Biometry and Environmental System Analysis, University of Freiburg, Tennenbacherstr. 4, 79106 Freiburg, Germany

**Keywords:** Species richness, Internal validity, External validity, Silvoarable, Silvopasture

## Abstract

In our article ‘European agroforestry has no unequivocal effect on biodiversity: a time-cumulative meta-analysis’ (BMC Ecology and Evolution, 2021) we synthesize the effect of agroforestry on biodiversity. Boinot et al. (BMC Ecology and Evolution, 2022) criticise our approach arguing that our definitions of agroforestry and biodiversity are too narrow; that we use inappropriate control sites for primary studies lacking distance to the treatment sites; that there are too few studies for a meta-analysis in silvoarable systems; and that local practice should be emphasized. We agree on the importance of local practices and that more studies would improve the robustness of our meta-analytical results. However, our conclusions are robust to removing studies criticised for inappropriate controls. We also recognize the problem of different definitions of agroforestry and using species richness as sole proxy for biodiversity. We appreciate being given the opportunity to clarify our results and to trigger future discussions about definitions and the interpretation of results from meta-analysis.

**Supplementary Information:**

The online version contains supplementary material available at 10.1186/s12862-022-02016-7.

In our recent meta-analysis [1], we have shown that agroforestry has no overall positive effect on biodiversity, but we found an effect of small magnitude comparing agroforestry with cropland. In a comment, Boinot et al. [2] raised concerns about our definitions of agroforestry and biodiversity, the selection of the control sites used in our meta-analysis and the applicability of our results for conservation. We are grateful for stimulating a discussion about research quality in agroforestry and respond in the following to each of the points raised.

## Hedges and agroforestry

Agroforestry is a land-use system combining agriculture or pasture with woody elements. Some definitions emphasize a mixture of trees with crops and/or animals on the same land and a combined production system [3, 4], while others also talk about agroforestry if trees and hedges are grown only at the border of crop fields and pastures [5]. Boinot et al. [2] especially argue for including hedges grown next to fields in the definition of agroforestry, which we did not cover with our meta-analysis. Today, hedges are generally not cultivated for wood production. Even in the example cited by Boinot et al., the majority of farmers stated to plant hedges for conservation and cultural purposes [2, 6]. Hedges offer habitat to different taxonomic groups and we would welcome a formal meta-analysis on the effect of hedges in different landscapes, in addition to existing reviews [7–9]. For our meta-analysis, we have chosen the narrower definition of agroforestry, only including systems with alternating trees and crops/pastures under the same management, to disentangle landscape structural elements and actual production systems.

## Species richness as proxy for biodiversity

We agree with Boinot et al. that biodiversity has more dimensions than species richness alone. However, species richness is one very important aspect of biodiversity and showed to be related to abundance and biomass [10, 11]. It is further the measure for biodiversity most commonly used in the literature, thus best suited for including many studies in a meta-analysis. We want to emphasize that our main objective was not to provide a comprehensive conservation recommendation covering all potentially relevant aspects of biodiversity. We explicitly state in our article that we show the effects on biodiversity proxied predominantly by species richness.

Boinot et al. state that 22 of 28 studies found some effects of agroforestry on abundance, species composition or functional groups and hence our conclusion about biodiversity are not valid. Although we assume that their point was to emphasize the multidimensionality of biodiversity, we want to draw attention to the danger of such an argument. Vote counting is a statistically flawed and unreliable research synthesis method [12]. We have selected species richness as the most commonly reported measure of biodiversity, and hence most suitable for meta-analysis. Not having found an effect on one measure of biodiversity, i.e. species richness, and searching for another until one may be “significantly” positively affected, would constitute a case of p-hacking and thus an unacceptable scientific practice [13]. Others are welcome to extend our analyses to other measures, although we do not expect different results for abundance, functional or phylogenetic diversity, as they are typically highly correlated with species richness.

## Control site selection

**Fig. 1:**
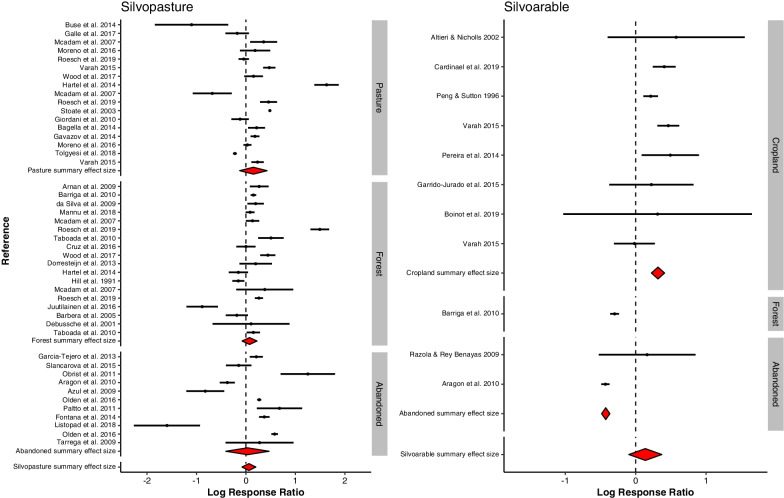
Meta-analysis of a subset of studies from [1], excluding studies argued by [2] to have control locations too close to the treatment sites. Silvopasture was reduced by three effects sizes and silvoarable reduced from 17 to 11 effect sizes

Boinot et al. criticise that some of our case-control comparisons on which the calculation of effect sizes is based, underestimate the effect of agroforestry on biodiversity. Indeed, we have included studies for which control locations were close to the treatment sites and this may potentially underestimate the effect of agroforestry on biodiversity. We have tested whether this was the case by excluding the sites that Boinot et al. regard as inappropriate [for code and data see Additional file 1 and 2]. The conclusion from the meta-analysis did not change after discarding the case-control comparisons criticized (Fig. 1).

## Meta-analysis and local practices

For meta-analyses, a minimum of primary studies is required to compute a meaningful summary effect [14]. Boinot et al. point out that it is too early for a meta-analysis on alley cropping agroforestry systems given that there are few studies with an unbalanced sampling of age and taxonomic groups. As we have not computed a summary effect size for alley cropping and it is not even an existing category in our meta-analysis, we assume that silvoarable systems are meant here. We agree that too few studies in silvoarable systems exist for each age and taxonomic group to derive very robust conclusions.

However, in the same breath, Boinot et al. conclude that our meta-analysis ‘most likely underestimates the positive effects’. We wonder about such a conclusion on ‘likely positive effects’ that are necessarily based on the same few primary studies that are said to be too few for meta-analysis. In the absence of good primary studies, no general conclusions can be drawn in either direction and it is unjustified to assume that there must be a strong positive effect of agroforestry on biodiversity. We however think that even with a low number of primary studies, a quantitative synthesis in form of a meta-analysis will outcompete the statement of each individual study and allow more general and evidence-based conclusions [15]. Thus a systematic research synthesis increases the evidence base, even by including only few effect sizes [15]. We also agree that there is room for improvement and welcome future and complementary efforts on different aspects of land use, biodiversity and by increasing the number of primary studies.

The effect of agroforestry on biodiversity may be influenced by local differences as Boinot et al. point out. We agree that local differences should be considered, if they have an influence on the impact of agroforestry on biodiversity. We want to remind that a fundamental idea of evidence-based practice is the identification of a causal link between an impact, e.g. a conservation measure, and an outcome of interest [16]. These direct links are difficult to determine if unmeasured and locally differing variables additionally influence the outcome of interest, which is often the case in ecology. Consequently, the transferability of study results from one local context to another is not straightforward. Nevertheless, in the absence of knowledge about the local conditions under which agroforestry may be or not be beneficial for biodiversity, a meta-analysis across many local contexts synthesising systematically searched literature, is the best and most generalisable evidence we can have.

## Supplementary Information

**Additional file 1.**Data for the meta-analysis on biodiversity.

**Additional file 2.** Description of data tables and R-Code of the analysis.

## Data Availability

All data required for the replication of the analysis are available in the supplementary material.
