# Supplementary material for: Reply to: “Research on agroforestry systems and biodiversity conservation: what can we conclude so far and what should we improve?” by Boinot et al. 2022
Source: BMC Ecol Evol. 2022 May 18;22:65. doi: 10.1186/s12862-022-02016-7 (PMC9115991; doi:10.1186/s12862-022-02016-7)
Supplement: Supplementary file 2 — Additional file 2. Description of data tables and R-Code of the analysis. [file 12862_2022_2016_MOESM2_ESM.zip › Supplementary_Response_Boinot_et_al.html]

Agroforestry Meta Analyses


# Agroforestry Meta Analyses

#### Anne Mupepele

#### 3/21/2022

This code was created in R version 4.0.2 (2020-06-22) with package versions up to date in July 2020.

# Macros

## Define colours

```
# define colours for different taxonomic groups
arth.col <- "#173F5F"
bat.col <- "#20639B"
plant.col <- "#3CAEA3"
bird.col <- "#F6D55C"
flb.col <- "#f28875"  # old red colour "#ED553B"
```

## Define functions

Combined standard deviation (GSD) (Square root of the ‘pooled variance’):

s=\(\sqrt{\dfrac{\displaystyle\sum\_{i=1}^{k}{(n\_i-1)s\_i^2+n\_i(\bar{x}\_i-\bar{x})^2}}{(\displaystyle\sum\_{i=1}^{k}{n\_i})-1}}\) (Duncan K, 1979 in Mauri Ora 7:139-142)

```
GSD <- function(means,sds,ns){sqrt((sum((ns-1)*sds^2+ns*(means-mean(means))^2))/(sum(ns)-1))}
means <- c(4,5)
sds <- c(1,3)
ns <- c(10,11)
```

Highlighting y-axis label in ggplot to make summary effect size labels bold.

```
highlight <- function(x,y){
  exp <- vector(length = 0, mode = "expression")
  for (i in seq_along(x)) {
    if (i %in% y) exp[[i]] <- bquote(bold(.(x[i])))
    else exp[[i]] <- x[i]
  }
return(exp)
}
```

Extract the legend from ggplot to provide one legend for several plot

```
g_legend<-function(a.gplot){ 
  tmp <- ggplot_gtable(ggplot_build(a.gplot))
  leg <- which(sapply(tmp$grobs, function(x) x$name) == "guide-box")
  legend <- tmp$grobs[[leg]]
  return(legend)}
```

# Data

## Datasets

- ‘biodivdat’ are the raw data (86 rows and 29 columns) loaded from Meta\_Anal\_DataR\_Biodiv\_v4.csv
  - Version 4: includes the latest literature search from February 2020
  - Previous versions: Version 1: 2016-2018 Version 2: 2016-2019 are used in Agroforestry\_MetaAnalysis\_v2.Rmd
- ‘ef’ includes the cleaned raw data (cleaned by e.g. restructured factor levels) and the effect sizes (log response ratio). (87 rows and 36 columns) one row reflects a unique combinations of agroforestry system (silvoarable or silvopasture), control type (forest, agricultural fields, pastures or abandoned and hence shrubby agroforestry systems) and taxonomic groups (*resolution as high as possible, e.g. one effect size for bees, one for butterflies*)
- ‘ef1’ includes the cleaned raw data and the effect sizes (log response ratio). (69 rows and 35 columns) one row reflects a unique combinations of agroforestry system (silvoarable or silvopasture), control type (forest, agricultural fields, pastures or abandoned and hence shrubby agroforestry systems) and taxonomic groups (*low resolution: arthropods, birds, bats, plants, FLB (fungi, lichen plus bryophytes)*)
- ‘s’ are the data per study, i.e. one study = one row

Columns and their description

- Assessor - name of the author who has extracted the information from the study
- AuthorSnip - key for the primary studies where data were extracted from
- Title - title of the primary studies where data were extracted from
- Climate - climate zone according to Walter1991
- Climate1 - climate zone merging alpine studies with ‘temperate’ and atlantic studies (‘L’) with Mediterranean.
- Country - country in which the primary study site was located
- Agroforestry\_System - distinguishing slivopastoral systems from silvoarable systems
- Study\_design\_Anne - study design in the primary study. One of: Case-Control, Before-After, Observational
- FinalLoE - the level of evidence according to the evidence assessment Mupepele2016a
- Comparator - the ‘control type’ with which the agroforestry system was compared to calculate the effect size in the meta-analysis.
- Dominant\_Tree\_Species - the dominant tree species in the agroforestry system
- Biodiversity\_Measure - the quantifiable measuer that was used to represent biodiversity. One of: species richness, family richness (number of families as mostly arthropods were not identified to species level), shannon diversity or log-series.
- Biodiversity\_Group - the taxonomic group investigated, reported as it was given in the primary study
- Biodiversity\_Group1 - restructuring the taxonomic levels into one of: ‘Arthropods’, ‘Bats’, ‘Birds’, ‘Plants’ or ‘Fungi, Lichen and Bryophytes’
- Sampling\_Method - approach used to sample biodiversity, e.g. transects or pan-traps
- agrof\_mean agrof\_sd agrof\_n - Mean, standard deviation (sd) and sample size of biodiversity measured in agroforestry field(s)
- control\_mean control\_sd control\_n - Mean, standard deviation (sd) and sample size of biodiversity measured in the control site(s), which is specified in the columns ‘Comparator’.
- Sampling\_Year\_Start - the year in which the sampling took place or started
- Sampling\_Year\_End - the year in which the sampling ended if several years were sampled
- Comments - additional information on personal communication with authors or missing standard deviation and transformations.

```
biodivdat <- read.csv2("12862_2022_2016_MOESM1_ESM.csv",dec=".",sep=",",stringsAsFactors=T)
```

## Process data

Impute missing values and restructure category levels to create ‘ef’.

```
#Imputing missing standard deviation: Replace NA in the two column with the standard deviations with the mean across this column.
biodivdat$agrof_sd <- replace(biodivdat$agrof_sd,is.na(biodivdat$agrof_sd),mean(biodivdat$agrof_sd,na.rm=T))
biodivdat$control_sd<- replace(biodivdat$control_sd,is.na(biodivdat$control_sd),mean(biodivdat$control_sd,na.rm=T))

#Restructure category levels
biodivdat$Biodiversity_Group1  <- factor(car::recode(biodivdat$Biodiversity_Group,"c('Ants','Arthropods', 'Bees','Beetles','Bombus','Butterflies','Carabid','Collembola','Insects','Scarabaeoidea','Solitary Bees','Spiders','Staphylinid','Isopoda','Orthoptera and Mantidae','Diplopoda','Earthworms')='Arthropods';c('Birds','Passerine birds', 'Woodpeckers')='Birds';c('Plants','Vascular plants','Shrubs')='Plants';c('Fungi','Lichen','Bryophytes')='FLB'")) #Plants are now all vascular plants
biodivdat$Country <- factor(car::recode(biodivdat$Country,"c('UK-Northern Ireland', 'UK-Scotland', 'UK-Wales')='UK'"))
biodivdat$Dominant_Tree_Species <- gsub("([A-Za-z]+).*", "\\1", biodivdat$Dominant_Tree_Species)
biodivdat$Publication_Year <- as.numeric(str_extract_all(biodivdat$AuthorSnip, "[0-9]+"))
biodivdat$Climate1 <- factor(car::recode(biodivdat$Climate,"'L'='M';'Alpine'='N'"))
biodivdat$Comparator <- car::recode(biodivdat$Comparator,"'Forest_Plantation'='Forest'")
#Impute the missing sampling year by using the publication year
biodivdat$Sampling_Year_Start[is.na(biodivdat$Sampling_Year_Start)] <-  str_extract(biodivdat$AuthorSnip, "\\-*\\d+\\.*\\d*")[is.na(biodivdat$Sampling_Year_Start)]
biodivdat$Sampling_Year_Start <- as.numeric(biodivdat$Sampling_Year_Start)
biodivdat$LoEsc <- as.numeric(as.character(car::recode(biodivdat$FinalLoE,"'LoE2b'=2.3;'LoE3a'=3;'LoE3b'=3.3;'LoE4a'=4;'LoE4b'=4.3")))
```

Create a data frame with log response ratio that will be used as effect size in the meta-analysis.

```
ef<-escalc(measure = "ROM", 
           m1i = agrof_mean, sd1i = agrof_sd, n1i = agrof_n, 
           m2i = control_mean, sd2i = control_sd, n2i = control_n, 
           data = biodivdat)
```

Combine effect sizes to have one effect size per combination of study, taxonomic group (according to Biodiversity\_Group1, i.e. 5 levels), agroforestry system and control type (comparator). Studies such as Varah 2015 have an effect size for butterflies and bees, which are both ‘arthropods’ and will be combined.

```
table(duplicated(ef[,names(ef)%in%c("AuthorSnip","Agroforestry_System", "Comparator","Biodiversity_Group1")])) #will remain with only 68 effect sizes
```

```
## 
## FALSE  TRUE 
##    69    14
```

```
ef$test <- paste(ef$AuthorSnip,ef$Agroforestry_System,ef$Comparator,ef$Biodiversity_Group1)
df <- data.frame(test=character(),
                agrof_mean_comb=numeric(),
                agrof_sd_comb=numeric(),
                agrof_n_comb=integer(),
                control_mean_comb=numeric(),
                control_sd_comb=numeric(),
                control_n_comb=integer(),
                stringsAsFactors=FALSE)#stringsASFactors= F to avoid that characters are converted to factors! This is the new default in R4.0.2

for(i in 1:as.numeric(table(duplicated(ef$test))[2])){                
tomerge <- ef[ef$test==ef$test[duplicated(ef$test)][i],]
df[i,1] <- tomerge$test[1]
df[i,2] <- mean(tomerge$agrof_mean)
df[i,3] <- GSD(tomerge$agrof_mean,tomerge$agrof_sd,tomerge$agrof_n)
df[i,4] <- sum(tomerge$agrof_n)
df[i,5] <- mean(tomerge$control_mean)
df[i,6] <- GSD(tomerge$control_mean,tomerge$control_sd,tomerge$control_n)
df[i,7] <- sum(tomerge$control_n)
}

dfchange <- df[duplicated(df$test)==F,]

efn <- ef[duplicated(ef$test)==F,] #throw the duplicates out of the full data set
#replace the values by new values

bn <- merge(efn,dfchange,by="test",all=T) #merge the old and the new data
bn$agrof_mean[is.na(bn$agrof_mean_comb)==F] <- bn$agrof_mean_comb[is.na(bn$agrof_mean_comb)==F]

ef1<-escalc(measure = "ROM", 
           m1i = agrof_mean, sd1i = agrof_sd, n1i = agrof_n, 
           m2i = control_mean, sd2i = control_sd, n2i = control_n, 
           data = bn[,2:34])

#data.frame(ef1$yi,efn[order(efn$AuthorSnip),]$yi,efn[order(efn$AuthorSnip),]$AuthorSnip,ef1$AuthorSnip) # to verify whether the new data frame matches the unchanged rows of the previous dataset 'efn'.
```

Create a data frame with one data entry (row) per study, instead of one per effect size, to analyse how many studies from one country are included etc.

```
s <- ef1 %>% distinct(AuthorSnip, .keep_all = TRUE)
table(s$Country[s$Climate1=="N"])
```

```
## 
##     Belgium     Finland      France     Germany       Italy    Portugal 
##           1           0           3           2           1           0 
##     Romania       Spain      Sweden Switzerland      Turkey          UK 
##           4           0           0           2           0           6
```

```
table(s$Climate1)
```

```
## 
##  B  M  N 
##  4 27 19
```

# Reply Boinot et al. 2022

Additional analysis excluding all observational studies and those that Boinot claimed (L78) that the control sites are too nearby:

```
critByBoinot <- c(names(table(droplevels(ef1[ef1$Study_design!="Case-Control",]$AuthorSnip))),"Peng et al. 1993","Akbulut et al. 2003","Lopez-Sanchez et al. 2016a")
ef_subBoinot <- droplevels(ef1[!ef1$AuthorSnip%in%critByBoinot,])
ef_subBoinot$Comparator <- car::recode(ef_subBoinot$Comparator,"'Agriculture'='Cropland'")
```

I’ll first combine the two figures with silvopasture and silvoarable and add the overall effect size

## Silvopasture subgroup forest plot

```
rma_intercept <- rma.mv(yi, vi, data = ef_subBoinot, method = "ML",random = list(~ 1 | AuthorSnip))

#Silvopasture
dat1 <- droplevels(ef_subBoinot[ef_subBoinot$Agroforestry_System=="Silvopasture",][,names(ef_subBoinot)%in%c("AuthorSnip","Comparator","Biodiversity_Group1","yi","vi")])
dat1$ci.lb <- summary(dat1)$ci.lb
dat1$ci.ub <- summary(dat1)$ci.ub
dat1$Type <- as.factor(rep("EffectSize",nrow(dat1)))

rma_silvopasture <- rma.mv(yi, vi, data = dat1, method = "ML",random = list(~ 1 | AuthorSnip))
model <- vector("list",length(levels(dat1$Comparator))) #empty list of length() elements
for (i in 1:length(levels(dat1$Comparator))){ 
  model[[i]] <- rma.mv(yi,vi,subset=(Comparator==levels(Comparator)[i]),data=dat1, method="ML",random=list(~1|AuthorSnip))
}
names(model) <- levels(dat1$Comparator)

dat2 <- data.frame(AuthorSnip=paste(levels(dat1$Comparator),rep(" summary effect size",3),sep=""),Comparator=levels(dat1$Comparator),Biodiversity_Group1=rep("",3),yi=sapply(1:3,function(i)as.numeric(model[[i]]$beta)),vi=as.numeric(rep(NA,3)),ci.lb=sapply(1:3,function(i)as.numeric(model[[i]]$ci.lb)),ci.ub=sapply(1:3,function(i)as.numeric(model[[i]]$ci.ub)),Type=rep("Summary",3))

dat3 <- data.frame(AuthorSnip=rep("Silvopasture summary effect size",1),Comparator=rep("",1),Biodiversity_Group1=rep("",1),yi=as.numeric(rma_silvopasture$beta), vi=as.numeric(rep(NA,1)),ci.lb=as.numeric(rma_silvopasture$ci.lb),ci.ub=as.numeric(rma_silvopasture$ci.ub),Type=rep("GrandSummary",1))


dat1 <- lapply(dat1, function(x) if(is.factor(x)) as.character(x) else x)
dat2 <- lapply(dat2, function(x) if(is.factor(x)) as.character(x) else x)
dat3 <- lapply(dat3, function(x) if(is.factor(x)) as.character(x) else x)

dat <- rbind(data.frame(dat1),data.frame(dat2),data.frame(dat3))
dat$Comparator <- factor(dat$Comparator,levels=c("Pasture","Forest","Abandoned"))

dat <- dat[order(factor(dat$Type,level=c("EffectSize","Summary","GrandSummary")),factor(dat$Comparator,level=c("Pasture","Forest","Abandoned")),factor(dat$Biodiversity_Group1)),]

# add numbered label for order of studies
dat$label <- paste(nrow(dat):1+10) 

# add multiple as biodiversity group category for summary effect size
dat$Biodiversity_Group1 <- factor(dat$Biodiversity_Group1,levels = c("Arthropods","Bats","Birds", "FLB", "Plants", ""))
levels(dat$Biodiversity_Group1)[levels(dat$Biodiversity_Group1) == ""] <- "Multiple"

# remove NA for grey box of overall effect
dat$Comparator <- factor(dat$Comparator, levels = levels(addNA(dat$Comparator)), labels = c(levels(dat$Comparator), " "), exclude = NULL) 

# define colours
cols_neutral = c(rep("black",4), "grey")

#data for diamonds as summary effect size
sub <- dat[dat$Type%in%c("Summary","SilvopastureSummary","GrandSummary"),]
diamonds <- data.frame(
  x=c(sapply(1:4,function(i)c(sub[i,]$ci.lb,sub[i,]$yi,sub[i,]$ci.ub,sub[i,]$yi))),
  y=rep(c(1,0.6,1,1.4),4),
  Comparator=factor(rep(levels(sub$Comparator),each=4)),Biodiversity_Group1=factor(rep("Multiple",16),levels = c("Arthropods","Bats","Birds", "FLB", "Plants", "Multiple")))

# plot
silvopasture_forest <- ggplot(dat, aes(y=label, x=yi, xmin=ci.lb, xmax=ci.ub)) +
  geom_vline(xintercept=0, color='black', linetype='dashed')+   #Add a vertical dashed line indicating an effect size of zero, for reference
  geom_errorbarh(size=0.8,position=position_dodge(width = 0.5), alpha=1,height=0) +
geom_point(data=subset(dat, Type=='EffectSize'),size=0.8,stat= "identity", color = 'black')+ #Add data points and color them black
  geom_point(data=subset(dat, Type!='EffectSize'), color='white')+ #Add 'special' points for the summary estimates
  geom_errorbarh(data=subset(dat, Type!='EffectSize'),size=0.9,position=position_dodge(width = 0.5), alpha=1,height=0,color='white')+
  geom_polygon(data = diamonds,aes(x = x, y = y),fill="red",color="black",inherit.aes=F)+ #add summary diamond
  scale_x_continuous(limits=c(-2.3,2.3), name='Log Response Ratio')+ #Specify the limits of the x-axis and relabel it to something more meaningful
  scale_y_discrete(breaks = dat$label, labels = highlight(as.character(dat$AuthorSnip),c(53,54,55,56)))+ # numbers refer to row numbers of dat
  ylab('Reference')+ #Give y-axis a meaningful label
 facet_grid(Comparator~., scales= 'free', space='free')+ #Create sub-plots (i.e., facets) based on levels of Comparator, I could change label here ,labeller=hospital_labeller. And allow them to have their own unique axes (so authors don't redundantly repeat)
  ggtitle("Silvopasture")+
 scale_fill_manual(values=cols_neutral, guide = F)+
 #scale_color_manual(values=cols6, name = "Taxonomic group", labels=c("Arthropods","Bats","Birds", "Fungi, Lichens,\n& Bryophytes", "Plants", "Multiple"))+ # legend title
  theme_bw()+
  theme(panel.grid.major=element_blank(),
        panel.grid.minor=element_blank(),
        panel.border=element_blank(),
        axis.line=element_line(),
        axis.text.y = element_text(size=7),
        axis.title = element_text(size=8,face ="bold"),
        strip.background = element_rect(colour="white", fill="white"), #strip refers to the facet labels, but can only change them all at ones.
        legend.position='right')

#remove the grey background on the last facet label that characterizes only the silvoarable summary effect size
g1 <- ggplot_gtable(ggplot_build(silvopasture_forest+theme(legend.position="none"))) #create gtable from modified (without legend) silvopasture_forest ggplot
stripr <- which(grepl('strip-r', g1$layout$name))
fills <- c("grey","grey","grey","white")
k <- 1
for (i in stripr) {
j <- which(grepl('rect', g1$grobs[[i]]$grobs[[1]]$childrenOrder))
g1$grobs[[i]]$grobs[[1]]$children[[j]]$gp$fill <- fills[k]
k <- k+1
}
```

## Silvoarable subgroup forest plot

```
rma_intercept <- rma.mv(yi, vi, data = ef_subBoinot, method = "ML",random = list(~ 1 | AuthorSnip))

#Silvoarable
dat1 <- droplevels(ef_subBoinot[ef_subBoinot$Agroforestry_System=="Silvoarable",][,names(ef_subBoinot)%in%c("AuthorSnip","Comparator","Biodiversity_Group1","yi","vi")])
dat1$ci.lb <- summary(dat1)$ci.lb
dat1$ci.ub <- summary(dat1)$ci.ub
dat1$Type <- as.factor(rep("EffectSize",nrow(dat1)))

rma_silvoarable <- rma.mv(yi, vi, data = dat1, method = "ML",random = list(~ 1 | AuthorSnip))
model <- vector("list",length(levels(dat1$Comparator))) #empty list of length() elements
for (i in 1:2){ 
  model[[i]] <- rma.mv(yi,vi,subset=(Comparator==levels(Comparator)[i]),data=dat1, method="ML",random=list(~1|AuthorSnip))
}
names(model) <- levels(dat1$Comparator)

dat2 <- data.frame(AuthorSnip=paste(levels(dat1$Comparator),rep(" summary effect size",3),sep=""),Comparator=levels(dat1$Comparator),Biodiversity_Group1=rep("",3),yi=c(sapply(1:2,function(i)as.numeric(model[[i]]$beta)),"NA"),vi=as.numeric(rep(NA,3)),ci.lb=c(sapply(1:2,function(i)as.numeric(model[[i]]$ci.lb)),"NA"),ci.ub=c(sapply(1:2,function(i)as.numeric(model[[i]]$ci.ub)),"NA"),Type=rep("Summary",3))

dat3 <- data.frame(AuthorSnip=rep("Silvoarable summary effect size",1),Comparator=rep("",1),Biodiversity_Group1=rep("",1),yi=as.numeric(rma_silvoarable$beta),vi=as.numeric(rep(NA,1)),ci.lb=as.numeric(rma_silvoarable$ci.lb),ci.ub=as.numeric(rma_silvoarable$ci.ub),Type=rep("GrandSummary",1))

dat1 <- lapply(dat1, function(x) if(is.factor(x)) as.character(x) else x)
dat2 <- lapply(dat2, function(x) if(is.factor(x)) as.character(x) else x)
dat3 <- lapply(dat3, function(x) if(is.factor(x)) as.character(x) else x)

dat <- rbind(data.frame(dat1),data.frame(dat2)[1:2,],data.frame(dat3))
dat$Comparator <- factor(dat$Comparator,levels=c("Cropland","Forest","Abandoned"))

dat <- dat[order(factor(dat$Type,level=c("EffectSize","Summary","GrandSummary")),factor(dat$Comparator,level=c("Cropland","Forest","Abandoned")),factor(dat$Biodiversity_Group1)),]

# add label to order studies
dat$label <- paste(nrow(dat):1+10) 

# add multiple as biodiversity group category for summary effect size
dat$Biodiversity_Group1 <- factor(dat$Biodiversity_Group1,levels = c("Arthropods","Birds", "FLB", "Plants", ""))
levels(dat$Biodiversity_Group1)[levels(dat$Biodiversity_Group1) == ""] <- "Multiple"

# remove NA for grey box of overall effect
dat$Comparator <- factor(dat$Comparator, levels = levels(addNA(dat$Comparator)), labels = c(levels(dat$Comparator), " "), exclude = NULL) 

# define colours
cols = c(arth.col, bird.col, flb.col, plant.col, "grey") # no bat studies in silvoarable - one colour less

#dat[dat$AuthorSnip=="Forest summary effect size",]$yi <- 0.39332687
#dat[dat$AuthorSnip=="Forest summary effect size",]$vi <- NA
#dat[dat$AuthorSnip=="Forest summary effect size",]$ci.lb <- 0.06542608
#dat[dat$AuthorSnip=="Forest summary effect size",]$ci.ub <-  0.72122765
#dat <- dat[!dat$AuthorSnip=="Forest summary effect size",]
         
str(dat)
```

```
## 'data.frame':    14 obs. of  9 variables:
##  $ AuthorSnip         : chr  "Altieri & Nicholls 2002" "Cardinael et al. 2019" "Peng & Sutton 1996" "Varah 2015" ...
##  $ Comparator         : Factor w/ 4 levels "Cropland","Forest",..: 1 1 1 1 1 1 1 1 2 3 ...
##  $ Biodiversity_Group1: Factor w/ 5 levels "Arthropods","Birds",..: 1 1 1 1 2 3 4 4 1 2 ...
##  $ yi                 : chr  "0.575364144903562" "0.405465108108164" "0.213574100298059" "0.463572738915445" ...
##  $ vi                 : num  0.24696 0.00691 0.00287 0.00629 0.04306 ...
##  $ ci.lb              : chr  "-0.398645722476754" "0.242569387943101" "0.108598083986496" "0.30807684072932" ...
##  $ ci.ub              : chr  "1.54937401228388" "0.568360828273228" "0.318550116609622" "0.61906863710157" ...
##  $ Type               : chr  "EffectSize" "EffectSize" "EffectSize" "EffectSize" ...
##  $ label              : chr  "24" "23" "22" "21" ...
```

```
dat$yi <- as.numeric(dat$yi)
dat$ci.lb <- as.numeric(dat$ci.lb)
dat$ci.ub <- as.numeric(dat$ci.ub)


#data for diamonds as summary effect size
sub <- dat[dat$Type%in%c("Summary","GrandSummary"),]
diamonds <- data.frame(
  x=c(sapply(1:3,function(i)c(sub[i,]$ci.lb,sub[i,]$yi,sub[i,]$ci.ub,sub[i,]$yi))),
  y=rep(c(1,0.8,1,1.2),3),
 Comparator=factor(rep(levels(sub$Comparator)[c(1,3,4)],each=4)),Biodiversity_Group1=factor(rep("Multiple",12),levels = c("Arthropods","Birds", "FLB", "Plants", "Multiple")))


# actual plot (from Dominic modified from Anne)
silvoarable_forest <- ggplot(dat, aes(y=label, x=yi, xmin=ci.lb, xmax=ci.ub)) +
  geom_vline(xintercept=0, color='black', linetype='dashed')+   #Add a vertical dashed line indicating an effect size of zero, for reference
  geom_errorbarh(size=0.8,position=position_dodge(width = 0.5), alpha=1,height=0) +
geom_point(data=subset(dat, Type=='EffectSize'),size=0.8,stat= "identity", color = 'black')+ #Add data points and color them black
  geom_point(data=subset(dat, Type!='EffectSize'), color='white')+ #Add 'special' points for the summary estimates
  geom_errorbarh(data=subset(dat, Type!='EffectSize'),size=0.9,position=position_dodge(width = 0.5), alpha=1,height=0,color='white')+
  geom_polygon(data = diamonds,aes(x = x, y = y),fill="red",color="black",inherit.aes=F)+ #add summary diamond
  scale_x_continuous(limits=c(-1.7,1.7), name='Log Response Ratio')+ #Specify the limits of the x-axis and relabel it to something more meaningful
  scale_y_discrete(breaks = dat$label, labels = highlight(as.character(dat$AuthorSnip),c(18,19,20,21)))+ # numbers refer to row numbers of dat
  ylab('Reference')+ #Give y-axis a meaningful label
  facet_grid(Comparator~., scales= 'free', space='free')+ #Create sub-plots (i.e., facets) based on levels of Comparator, I could change label here ,labeller=hospital_labeller. And allow them to have their own unique axes (so authors don't redundantly repeat)
  ggtitle("Silvoarable")+
 # scale_fill_manual(values=cols, guide = F)+
#  scale_color_manual(values=cols, name = "Taxonomic group", labels=c("Arthropods","Birds", "Fungi, Lichens,\n& Bryophytes", "Plants", ""))+ # legend title, empty field in label to remove 'multiple' from the legend
  theme_bw()+
  theme(panel.grid.major=element_blank(),
        panel.grid.minor=element_blank(),
        panel.border=element_blank(),
        axis.line=element_line(),
        axis.text.y = element_text(size=7),
        axis.title = element_text(size=8,face ="bold"),
        strip.background = element_rect(colour="white",fill=NA), #strip refers to the facet labels, but can only change them all at ones.
        legend.position='right')

#remove the grey background on the last facet label that characterizes only the silvoarable summary effect size
g2 <- ggplot_gtable(ggplot_build(silvoarable_forest+theme(legend.position="none",axis.title.y=element_blank()))) #create gtable from modified (remove legend and axis) silvoarable_forest ggplot
stripr <- which(grepl('strip-r', g2$layout$name))
fills <- c("grey","grey","grey","white")
k <- 1
for (i in stripr) {
j <- which(grepl('rect', g2$grobs[[i]]$grobs[[1]]$childrenOrder))
g2$grobs[[i]]$grobs[[1]]$children[[j]]$gp$fill <- fills[k]
k <- k+1
}
```

Translating the summary effect size of the Cropland subgroup analysis in the impact on biodiversity to estimate the magnitude of the effect.

```
exp(model$Cropland$beta)
```

```
##             [,1]
## intrcpt 1.373143
```

```
with(ef_subBoinot[ef_subBoinot$Comparator=="Cropland",],mean(control_mean))
```

```
## [1] 5.09625
```

### Combined forest plot

Combine the two subplots silvopasture and silvoarable and use a shared legend.

```
layoutmatrix <- rbind(c(1,1,2,2),
             c(1,1,2,2),
             c(1,1,2,2),
             c(1,1,2,2),
             c(1,1,2,2),
             c(1,1,2,2),
             c(1,1,2,2))


#cairo_pdf(file="Figure_Forest_Plot_SubForBoinot2022_v2.pdf",width =11,height =7)
grid.arrange(grobs=list(g1,g2),layout_matrix = layoutmatrix)
```

```
#dev.off()
```
